# Elicitor and Receptor Molecules: Orchestrators of Plant Defense and Immunity

**DOI:** 10.3390/ijms21030963

**Published:** 2020-01-31

**Authors:** Nurul Azmina Abdul Malik, Ilakiya Sharanee Kumar, Kalaivani Nadarajah

**Affiliations:** Department of Biological Sciences and Biotechnology, Faculty of Science and Technology, Universiti Kebangsaan Malaysia, 43600 Bangi, Selangor, Malaysia; azmina_malik93@yahoo.com (N.A.A.M.); ilakiya95sharanee@gmail.com (I.S.K.)

**Keywords:** plant defense systems, SAR, ISR, pathogen-associated molecular patterns (PAMPs), microbe-associated molecular patterns (MAMPs), herbivory-associated molecular patterns (HAMPs), damage-associated molecular patterns (DAMPs), plant pattern recognition receptors (PRRs), receptor molecules

## Abstract

Pathogen-associated molecular patterns (PAMPs), microbe-associated molecular patterns (MAMPs), herbivore-associated molecular patterns (HAMPs), and damage-associated molecular patterns (DAMPs) are molecules produced by microorganisms and insects in the event of infection, microbial priming, and insect predation. These molecules are then recognized by receptor molecules on or within the plant, which activates the defense signaling pathways, resulting in plant’s ability to overcome pathogenic invasion, induce systemic resistance, and protect against insect predation and damage. These small molecular motifs are conserved in all organisms. Fungi, bacteria, and insects have their own specific molecular patterns that induce defenses in plants. Most of the molecular patterns are either present as part of the pathogen’s structure or exudates (in bacteria and fungi), or insect saliva and honeydew. Since biotic stresses such as pathogens and insects can impair crop yield and production, understanding the interaction between these organisms and the host via the elicitor–receptor interaction is essential to equip us with the knowledge necessary to design durable resistance in plants. In addition, it is also important to look into the role played by beneficial microbes and synthetic elicitors in activating plants’ defense and protection against disease and predation. This review addresses receptors, elicitors, and the receptor–elicitor interactions where these components in fungi, bacteria, and insects will be elaborated, giving special emphasis to the molecules, responses, and mechanisms at play, variations between organisms where applicable, and applications and prospects.

## 1. Introduction

Plants are constantly threatened by an array of biotic stresses in their natural environment. Since plants are primary producers, they are at the bottom of most food chains and are prone to invasion or infection by bacteria, fungi, insects, and herbivores. Being sessile, they are compelled to defend themselves against a plethora of unavoidable stresses [1,2]. Although plants do not have an immune system as advanced as animals, plants are able to display resistance toward the diseases and damage caused by these organisms by relying on the innate immunity of cells and systemic signaling from the site of infection [3,4,5]. Infiltration of a host can happen either through stomata (natural opening), physical injuries, or direct penetration of the plant surfaces [6]. This two-way interaction involves the recognition of host invasion and manipulation of host biology to bring about the colonization of host or result in cellular damage [7,8,9,10]. Therefore, a successful plant defense mechanism should inhibit organismal infection, and limit insect and herbivore damage to the whole plant. 

The physical barrier in plants serves as first line of defense. This structural defense prevents pathogens and predators from invading the plant [11]. When infected, plants are able to recognize the changes in the cuticle, prompting activation of the defense responses [12]. Other mechanisms are activated when the immune responses in plants are induced due to infiltrations of either herbivores or pathogens. When pathogens and insects breach the first line of defense, it initiates a second line of defense that destroys the invading disease and pest by reducing structural damage beyond the site of infection and tissue damage. The second line of defense system is enhanced with the ability of plants to recognize certain pathogens and insects through their secretomes and other molecular patterns. These molecules interact with the plant surface, which amplifies the signals received through the transmission of plant signal molecules that induce signal transduction cascades and activate defense and resistance genes in resistant varieties, while the susceptible lines are overcome by the pathogens, insects, and herbivores [13,14]. Pathogens that infect the plants use different mechanisms based on their lifestyles that can either be biotrophic, necrotrophic, or hemibiotrophic [15,16]. While biotrophic pathogens feed off living tissue, the necrotrophic live off the dead, and the hemibiotrophic combine both strategies [17,18,19]. The plant too is divided into two systems based on its genetic basis of qualitative resistance (monogenic resistance) and quantitative resistance (polygenic resistance) [20]. The plant then reacts at the molecular level to invasion by the (i) non-host response, and (ii) host response depending on molecules secreted by both players [21,22]. As biotic stresses include insects, nematodes, and other hebivorous organisms, a breach of host defense provides for a different array of chemicals secretomes that reacts with the host and elicits a response that is mostly similar to what has been reported with pathogenic and non-pathogenic organisms.

Therefore, in this review, we will examine the roles played by elicitors, receptors, their interaction, and pathways activated in surmounting defense responses in hosts. These reactions involve responses to pathogens, non-pathogens, herbivores, and chemical signals. The existing gap in information and the potential applications of the knowledge acquired from this systematic review have been further elaborated.

## 2. Plant’s Immune Response: Key Components

To begin elucidation of the role of receptors and elicitors in defense, we first need to understand the immune system in plant systems. Plant immune responses can be divided into plant innate and plant-induced immunity. In both these immune systems, receptor and effector molecules play a crucial role in its activation and execution.

### 2.1. Plant Innate Immunity: Pre-Existing Defense

Plant innate immunity is composed of pathogen-associated molecular patterns (PAMP)-triggered immunity (PTI) followed by effector-triggered immunity (ETI), which perceives and protects plants from threatening pathogens [23,24,25,26]. Unlike an animal’s adaptive immune system, a plant’s innate immune system relies on pre-existing receptor molecules in the cells. Activation of the plant’s innate immunity is through the specific perception and recognition of PAMPs or microbe-associated molecular patterns (MAMPs) by pattern recognition receptors (PRRs) in plants (non-host resistance) or through identification by a resistance (R) protein-mediated process against race-specific effector molecules of pathogens (host resistance) [27,28]. In the case of non-host resistance, a particular plant can be resistant to different kinds of pathogens, whilst the same pathogens may infect other plant systems. This is a process initiated by the specific recognition of pathogen molecules called MAMPs by PRR-type membrane receptor proteins. In the first phase of plant innate immunity, PTI stops the colonization of pathogens after their PAMPs are recognized by PRRs in plants. PAMPs are evolutionarily conserved pathogen-derived molecules that constitute portions of fungal cell walls, structural components of pathogens, or flagellum [29]. Several PAMPs can be perceived by numerous plant species, although this is dependent on the plant species specificity and effectiveness [28,29,30]. Nonetheless, some PAMPs could only be recognized by a limited number of plant species receptor molecules [31,32,33,34,35,36,37]. PAMPs, herbivory-associated molecular patterns (HAMPs), and damage-associated molecular patterns (DAMPs) that are originally called endogenous elicitors are also referred to as MAMPs [38]. The recognition of MAMPs at the molecular level results in PAMP-triggered immunity (PTI), which causes reactive oxygen species (ROS) production, the activation of mitogen-activated protein kinases (MAPK), Ca^2+^ signaling, and ultimately transcriptional reprogramming [39,40].

There are various types of receptor molecules present in plants. The efficacy of these receptors are amazing, considering that with one receptor, a plant can recognize a complete taxonomic group that features a particular PAMP. For example, flagellin receptor FLS2 (*flg22*) enables the plant to recognize all mobile flagellated bacteria. However, it is possible for pathogens to avoid plant immunity through the mutation or deletion of these molecular patterns. A mutation in plants that affects the activity of PAMPs’ recognition causes the plants to be more susceptible to adapted pathogens and allows some disease development by non-adapting pathogens. Ve1 is an example of a plant receptor molecule in tomato, which binds to the Ave1 elicitor molecule from fungi. Ve1 can also be introduced as the PRR or R protein where the Ave1 peptide is referred to as the effector acting as MAMP [41,42,43].

Receptor-like kinases (RLKs) are types of PRRs located at transmembrane that possess extracellular domains that are essential in transmitting information from external stimuli by recognizing the ligands [6,44,45]. The RLK-mediated signal transmission of defense against pathogens responds to treatment with elicitors, pathogens, and signal molecules produced during biotic responses. The responses of RLKs are often dependent on specific ligands and pathogens. For instance, the S domain RLK (SFR1) is induced by bacteria and wounding, RLK3 is induced by oxidative stress and salicylic acid (SA), and potato receptor kinase (StPRKs)are induced by cell wall-degrading enzymes, short oligogalacturonides, and pathogen attack [46,47,48]. Further, wall-associated kinases (WAK) are induced by pathogens, SA, and cell wall pectin [49]. Some of these kinases have been identified as *R* genes. Recently a leucine-rich repeat (LRR) receptor was identified with the ability to bind bacterial flagellin, resulting in the activation of the defense pathway. These RLPs or RLKs activate specific plant defensive responses following their recognition [28]. While direct receptor–elicitor interaction has been demonstrated (as in the flagellin–FLS2 interaction), the complexity of this interaction still lacks understanding. Examples of receptor and elicitor molecules will be discussed in greater depth in the following sessions.

### 2.2. Plant-Induced Immunity: Effector Activated System

Equally important is the plant-induced immunity where signaling molecules activate the defense response to protect plant tissue from further damage from biotic or abiotic stresses [50,51,52]. In contrast to PTI, ETI involves effector molecules instead of PAMPs, and it is different from PAMPs, where effectors act as indicators of potential pathogens. Virulence factors from the pathogens are secreted into the plants and act as effector molecules. R proteins in the plants will identify the effectors to activate ETI [25,26,27]. After the R proteins in plants recognize its corresponding effectors, the plant immediately activates its immune responses through the induction of ETI [53]. ETI is induced in phase two of plant innate immunity. An example of the effector–*R* gene model in plant pathogen interaction can be seen in the interaction between coiled-coil, nucleotide binding site and leucine rich repeats (CC-NBS-LRR) of the Pi-ta receptor in rice and with the AvrPita effector of *Magnaporthe grisea* [54]. 

Most often when bacteria invade plants, EFR receptors that act as PRRs will detect the presence of the elongation factor (EF–Tu) elicitor molecules from the bacteria, which then triggers the immune system in plants. This form of immunity occurs in the cells’ post-R protein perception of effector proteins and the elicitation of oxidative burst, hormone accumulation, MAPK activation, antimicrobial production, and pathogenesis-related protein (PR protein) expression in response to the invasion [11,28,55,56,57]. The initiation of ETI results in the death of cells in the infected area and thus prevents infection from spreading to other parts of the plant, causing hypersensitive response (HR) [28], which results in programmed cell death (PCD) [58]. When the plant survives the infection in one site, it often develops increased resistance to subsequent attacks throughout the plant and enjoys protection against a wide range of pathogenic species. This phenomenon is known as systemic acquired resistance (SAR), where it is transmitted through the phloem to other parts of the plant via signal molecules, resulting in increased resistance throughout the plant [14,28,59].

Several examples of signaling molecules that are induced by pathogenic infection include SA, methyl salicylate, jasmonate (JA), and ethylene (ET). SA and ET/JA-mediated signaling pathways play a major role in plant resistance toward pathogens [60]. This results in higher levels of SA and its methyl ester, thus causing the production of PR proteins, including chitinases and other hydrolytic enzymes. Besides being an important factor in SAR, SA also plays a significant regulatory role in local HR against various pathogens. Methyl-SA [61], a methylated form of SA, is produced once the cell has been infected by the pathogen. This volatile SAR-inducing molecule travels to other parts of the plant through the plasmodesmata [62,63,64]. Fu et al. (2012) state that when SA is elevated at the area of infection, it will bind to the low-affinity binding receptor *NPR3* (Nonexpressor of PR Genes 3) and cause the degeneration of cell-death suppressor *NPR1*, which subsequently results in HR [65]. SA binds *NPR4*, a high-affinity receptor molecule in the cell to prevent the deterioration of *NPR1* and saves the cell [65]. Similarly, *NPR1* also takes part in SA and JA dependent defense pathways, thereby promoting appropriate immune response within the plant [66]. It has been shown that JA acts through a conserved signaling mechanism that has close resemblance to that described for other plant hormones such as auxin and gibberellins [67,68].

It has been shown that PAMPs such as lipopolysaccharides can induce SAR [69]. This system provides specific resistance to pathogens through the interaction of plant resistance (*R*) gene products and pathogen-derived avirulent gene products. A plant’s response to PAMPs can be viewed as an expression of basal resistance, since most of plant’s immunity is considered innate. Mitogen-activated protein kinase serves as a signal transducer that perceives the signal from the elicitor receptor interaction and thence amplifies and transmits the signal to distal tissues to activate the defense response systemically [70]. Signaling cascades generated by this response are responsible for the activation of plant’s innate immune system [27]. The above processes are in place to block the advancement of biotrophic organisms, which arrest the spread of infection to other host tissues through the production of ROS and nirogen dioxide (NO_2_) [20,71]. The necrotrophic pathogens on the other hand use the plant cell wall as the first line of defense, where they play a role in the integrity sensory system, leading to the activation of defense response and elevated levels of JA and ET [19]. The SA signaling pathway controls plant defense mechanisms against biotrophic pathogens while the ET/JA pathways are usually needed for plant resistance toward necrotrophic pathogens and herbivorous pests [72,73]. On the other hand, when an interaction occurs between plants and non-pathogenic microorganisms, systemic resistance [74] is activated. These microorganisms activate signaling pathways involving JA and ET, which trigger induced systemic resistance (ISR) throughout the plant. The colonization of these microorganisms initiates a signaling cascade throughout the plant and activates protective mechanisms. ISR results in enhanced resistance against pathogen attack rather than activating immediate defensive measures in the plant. Lipopolysaccharides (LPS) triggered by rhizobacteria are an example of molecules that are able to activate induced systemic resistance [74] against subsequent infections without the need for the accumulation of PR protein and phytoalexins [43,75].

## 3. Model of Interaction between Effector and *R* genes in Plant Immunity

### 3.1. The Guard Hypothesis

There have been other models that have materialized to explain the recognition process between a receptor molecule and elicitors besides the general recognition model. Amongst these models, the “Guard hypothesis” suggest that the R protein detects changes by the effector to the guard (host) protein. An example at hand for this model is the targeting of the RIN4 protein by the AvrRpm1 and Avrpt2 effectors, which cleaves the RIN4 in *Arabidopsis*, leading to its recognition by the R protein [76,77]. 

### 3.2. The Decoy Protein Hypothesis

Unlike the above, the “decoy protein” hypothesis is built on the concept that this decoy protein is able to mimic the pathogen effector target and restrict infection but has no direct role in immunity [77]. A good example for this model would be the decoying method of extracellular Protein-6 (Ecp6) produced by *Cladosporium fulvum* while infecting tomato that mimicks the chitin-binding capacity of the receptor to surpress chitin recognition by the host [78]. Based on the evolutionary basis upon which this hypothesis is built, this protein increases or decreases its affinity for effectors based on the presence or absence of the *R* gene [79,80]. 

### 3.3. The Zig-Zag Model

Finally, we approach the currently accepted zig-zag model, which basically involves interaction between the pathogen and the host. However, some scientists are of the opinion that this concept has oversimplified a complex process such as a pathosystem, while others question the terminologies and the specificity of the terms used [81,82]. However, there were some concerns highlighted with regard to this model. They are as follows. (1) The model does not include DAMPs in its prediction. (2) The model discounts the involvement of the environment in the interaction. (3) The model does not look into the possibility that the process may be random and not structured. (4) The model does not provide a timescale for the gain or loss of effectors. (5) The model does not provide details on the population context by which this gain or loss of effector takes place. (6) Finally, it lacks a qualitative model on the effects of miRNAs on the response of PTI and ETI (e.g., miR393, miR482) [8]. Therefore, while as a concept the zig-zag model does represent the interaction closely, the players and contributing factors need to be adjusted according to the pathosystem studied.

### 3.4. The Invasion and Multicomponent Model

In recent years, two more models have been put forth: the invasion and the multicomponent model. The invasion model is built on the zig-zag model and improves on its limitations. This model is like the zig-zag model except that the definition of the immunogenic molecules are different [61]. In this model, the molecules are seen as entities that play a role beyond just pathogenicity, therefore opening the molecule up to the effect of evolution and affecting the way these molecules interact and influence the pathogen–host fitness. The multicompent model, on the other hand, is different from other models where the *R* genes and effectors are described as independent where all plant pathogen-related information should be utilized to design techniques in plant breeding for resistance. This model is established on two main components: activation and modulation [83], which is further divided into (1) interaction between principle components (virulence targets and plant metabolism), (2) *R* gene activation of PRR-triggered signaling (PTS), (3) metabolic changes resulting in feedback regulation triggering hormone-tempered resistance (HTR), and (4) modulation of the resistance stage where PTS and the HTR together control resistance based on the pathogen’s lifestyle. However, at this juncture, we would like to state that these two recent models do not discredit the zig-zag model but highlight perspectives to be considered to provide a holistic view of plant immunity beyond molecular interaction.

## 4. The Plant Defense Response: The Players and Pathways Involved

**Figure 1:**
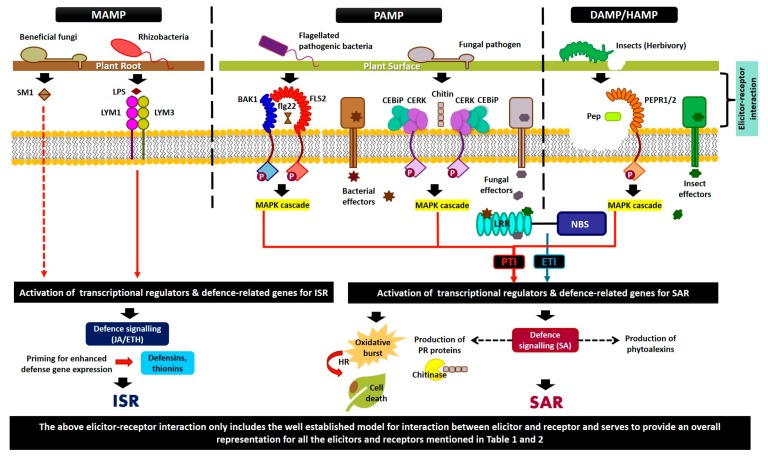
Pathogen-associated molecular patterns (PAMP)-triggered immunity (PTI) and effector-triggered immunity (ETI) for different types of molecular patterns produced by pathogenic and non-pathogenic microorganisms and insects (microbe-associated molecular patterns (MAMPs), damage-associated molecular patterns (DAMPs), and herbivore-associated molecular patterns (HAMPs). The well-established elicitor–receptor model for pathogenic fungus and bacteria, insects and non-pathogenic microbes are chitin elicitor binding protein/chitin elicitor receptor kinase (CEBIP/CERK), chitin, FLAGELLIN SENSITIVE2/BRI1-associated kinase (FLS2/BAK1), flg22, PEP receptor 1/2 (PEPR1/2), peptides (Pep), Lysin motif protein ½ (LYM1/LYM2), LPS, and hydrophobin-like elicitor (SM1). Other examples of elicitors and receptors are provided in Table 1 and Table 2.

The elicitor–receptor interaction triggers the pathway that sets the defense system in a plant into motion. A common early element of defense signaling is the modification of the ion permeability of the plasma membrane (Figure 1). Receptor molecule activation first stimulates the opening of ion channels across the membrane. Due to this, an influx of calcium ions into the plant cells occurs, which then activates reactive oxygen species (ROS) production in the cell. The accumulation of ROS around the location of proliferation has been reported in the fungal and bacterial pathogen infestation of plants [84]. The accumulation of ROS causes an oxidative burst that may act directly as a defense mechanism or signal the activation of other defenses [85]. These initial reactions are essential to initiate the signaling network that will activate all of the defense responses [86].

PCD initiated by pathogen attack involves the expression of a set of genes that organize the dismantling of cellular components and the quick accumulation of toxic molecules, thus depriving the pathogens of nutrients and ultimately resulting in cell death [87]. As a result, the rest of the plant remains unaffected due to HR [88]. It provides resistance against pathogens and is often preceded by the rapid accumulation of ROS. This oxidative burst stimulates and contributes to cell death and other defense mechanisms. It may also act to kill the pathogens directly [89]. There are two phases in ROS production: the first phase is the rapid, transient, nonspecific ROS production, and the second phase occurs later, where the concentration of ROS is much higher than the first [90].

PR proteins are produced as a consequence of SAR, and these defense-related proteins are closely associated with pathogen infection. An assortment of hydrolytic enzymes such as glucanases, chitinases, and other hydrolases are induced by fungal invasion. Together with the formation of PR proteins, plants also produce some proteins that inhibit herbivorous insect digestion as part of the plant defense mechanism. Proteins that interfere with herbivore digestion are among the diverse components of plant defense responses induced by JA. For example, some legumes synthesize α-amylase inhibitors that blocks the action of the starch-digesting enzyme α-amylase [91]. Anti-digestive proteins such as protease inhibitors are found in legumes, tomatoes, and other plants, where they halt protease activities [92]. After entering the herbivores’ digestive tract, the protease inhibitors obstruct protein digestion by binding tightly and specifically to the active site of the proteolytic enzymes. Insects that feed on plants containing protease inhibitors suffer reduced rates of growth and development. Plants that were transformed to accumulate increased levels of protease inhibitors suffered less damage from insect herbivores than untransformed control plants did [93]. 

Next, the synthesis of phytoalexins is initiated as a biochemical plant defense response. These molecules are considered to be the best-studied response of plants to bacterial and fungal invasion. Phytoalexins constitute a chemically complexed group of secondary metabolites with strong antimicrobial activity that accumulates around the site of infection and increases after pathogen attack. The production of phytoalexins is a regular mechanism of resistance toward pathogens in many plants. However, different plant families employ different types of secondary products as phytoalexins. For instance, isoflavonoids are common phytoalexins in leguminous plants such as soybean and alfalfa, whereas various sesquiterpenes are produced as phytoalexins, in solanaceous plants such as potato, tobacco, and tomato. Phytoalexins are generally undetectable in plants before infection, but they are synthesized very rapidly after pathogen attack. Experiments on genetically modified plants and pathogens have provided the first direct proof of phytoalexins’ function in vivo. For example, control plants without transgenes are less resistant than tobacco with a transgene that catalyzes the biosynthesis of phenylpropanoid phytoalexin resveratrol, which results in augmented resistance toward fungal pathogens [94]. In other experiments, pathogens transformed with genes encoding phytoalexin-degrading enzymes were able to infect plants, which were normally resistant [95]. Through these series of events, the defense mechanism is initiated and the message is transmitted to distal tissues to protect the plants. However, for the immune system and defense signaling to function, two key players are required: the elicitor and the receptor molecules. There are two steps in the elicitor receptor ligand interaction which fits into the address–message hypothesis proposed for the activation of receptor by neuropeptides [96], where the first step is the binding of elicitor to the receptor, resulting in its activation through the formation of a ligand receptor complex that results in conformational change. The second step involves the regulation of the receptor, which results in the transmission of the information received via transmembrane signaling [97]. Besides, the physiological effects and binding feature of the agonist/antagonist pairs correspond with the address–message concept. In the following sections, we look into various elicitors and receptors that are involved in responses to pathogens and insect predators and are responsible for receipt and thence amplification and transduction of the signal leading to defense activation.

## 5. The Elicitation of Defense: Elicitor Molecules, the Initiators of the Defense Cascade

Elicitor molecules do not have a signature structure and are made up of molecules such as oligosaccharides, peptides, lipids, and proteins. They include molecules released from or produced by pathogens [98,99] that provide information for plants to perceive and recognize signal from pathogens by cell surface-localized receptors, resulting in the activation of plant immunity [98]. One major research challenge in this area is the identification of receptors to elicitors, and to date, only a few receptors have been identified for specific elicitor molecules. Current reports have shown that plants are able to respond to the above array of elicitor molecules through the interaction with common elicitor motifs.

Elicitors in pathogens can be categorized into two categories: general elicitors and race-specific elicitors [100]. General elicitors are involved in the general resistance signaling pathways, while race-specific elicitors are associated with R gene-mediated signaling [101]. Elicitors that are capable of triggering defense in both non-host and host plants through the perceived presence of potential pathogens are known as general elicitors [41,102,103,104,105,106]. Most general elicitors are essentially present in pathogen cell walls as structural components e.g., glucan, chitin, flagellin, and lipopolysaccharides (LPS). Some general elicitors can only recognize a limited number of plants and are active only in specific hosts [74,107,108]. For instance, the oligosaccharides elicitors from *M. grisea* triggered phytoalexins synthesis in rice cells as the specific host and not in soybean cotyledon cells [107]. In contrast, β-glucan elicitors from *Phytophthora sojae* are able to activate defense responses in rice, soybean, and other non-hosts [109,110]. For example, a 75-kDa protein associated with the plasma membrane of *Glycine max* and *Phaseolus vulgaris* binds β-glucan elicitors from *Phytophthora* sp with high affinity. However, none of these proteins exhibit a signaling domain, suggesting that these β-glucan binding proteins may interact with other components to transduce the elicitor signal.

Meanwhile, race-specific elicitors play essential roles as virulence determinants [100]. These virulence determinants include harpins and *avr* gene products (see Table 1). This cultivar-specific (gene-for-gene) resistance can be determined by the pair of genes that complement each other between pathogen races and specific host cultivars [111]. The absence of either one of these genes in the interaction can result in disease [112,113,114,115,116,117]. In most plant pathogens, the initial elicitor and virulence determinant produced may be cell wall-degrading enzymes that facilitate infiltration. These enzymes provide the pathogen with nutrients and release pectic fragments that function as endogenous elicitors [108,118]. Elicitors are also produced in the leguminous–rhizobia interaction where the chemicals exuded by the plant will result in the recruitment of bacteria that orchestrate nodulation [119]. The lipo-chitooligosaccharides produced act as Nod factors and also provide evidence of the evolution of Nod factors from general elicitors [98,119]. Table 1 provides a list of elicitor molecules produced by bacteria, fungi, and insects.

### 5.1. Fungal Elicitor Molecules and their Ability to Induce Defense Response

An example of a fungal elicitor molecule is the chitooligosaccharide elicitor, chitin [28,59,120,121,122,123,124,125] (Table 1). Chitin is an important component of fungal pathogenicity, where pathogens with defects in chitin synthesis are significantly less virulent on susceptible hosts. Chitin, a polymer of *N*-acetyl-d-glucosamine, consists of (1–4)-linked 2-acetamido-2-deoxy-β-d-glucopyranose (GlcNAc) residues that are insoluble components of fungal cell walls [126]. It has been reported that the length of the *N*-acetylchitooligosaccharides affects the chitin-induced signaling activity. This can be shown by a lower activity of *N*-acetylchitooligosaccharides that is shorter than hexamers in activating defense response, where the highest induction of defense was seen with octaose and heptaose oligosaccharides ([GlcNAc] 7/8) in cultured-rice cells [127]. Chitin is recognized by plants, where it triggers a cascade of activities that leads to the elicitation of various defense responses which results in protection against fungal infections [37]. The expression of defense-regulating genes in rice suspension cells was induced by chitin [128,129,130,131] with increased resistance to rice blast [128]. In addition, chitin also induced ion efflux [132,133,134] 2013), the generation of ROS [128,135,136,137], increased levels of chitinases [138], higher levels of phytoalexins [139,140,141] and the induction of hypersensitivity in infected cells [126,128,142]. Chitinases that are commonly present in plants degrade chitin into soluble components [143] and thence trigger immunity. Furthermore, some toxins produced by pathogens are also able to function as elicitors at very low concentrations and elicit defense responses. For instance, the rice blast pathogen produces α-picolinic acid, which is a toxin that can elicit HR and increase disease resistance in rice by activating hydrogen peroxide generation and cell death [144].

Ethylene-inducing xylanase (EIX) is another classic example of a fungal elicitor. As the name suggests, this elicitor is often associated with the biosynthesis of ethylene, which leads to the leakage of electrolytes, the expression of PR proteins, and sometimes the manifestation of a hypersensitive response in some plant species. The corresponding receptors for this elicitor, namely *LeEix1* and *LeEix2*, were identified in tomato [145] and were shown to work as a duo whereby *LeEix1* attenuates the signals from *LeEix2* by acting as a decoy receptor. Cerebroside is a type of sphingolipid—an important component of the phytopathogen’s cell membrane—that demonstrates involvement in plant–pathogen interaction [146,147]. They are purported to induce the defense signal in rice and facilitate the synthesis of PR proteins and the production of H_2_O_2_ in tomato root tissues. In a study on *Fusarium* diseases, the induction of cerebrosides elevated the expression of PR proteins in tomato plants, although there was not any evidence of direct antifungal activity [147]. Further, plant MAMPs/PAMPs can also recognize β-1,3-glucan, which is another distinct type of fungal elicitor that constitutes the fungal cell wall [148]. The linear form of β-1,3-glucan known as laminarin (Lam) was shown to trigger defense-related responses in a wide number of plant species via the expression of ethylene-dependent and SA-dependent PR proteins [149]. Other types of fungal elicitors with corresponding plant systems are presented in Table 1 and Figure 1. 

### 5.2. Bacterial Elicitor Molecules and Their Ability to Induce Defense Response

Bacterial pathogens are capable of producing distinct categories of virulence factors that avoid PTI, advance growth, and increase the disease-causing ability to the host [150]. The delivery of virulence factors into plants is the most important element in bacterial pathogenesis [151] where it targets PRRs, represses PTI, and allows colonization of the bacteria [27]. After successful colonization, the bacteria use their extracellular virulence factors such as enzymes that degrade the cell wall and permeate the cell walls of plants to obtain water and nutrient supplies [152]. Type-III secreted effector (T3E) is an example of a virulence factor that is injected into the plant cells during bacterial invasion. By injecting T3E into the plant cells, the bacteria can either repress the plant defense or increase bacterial fitness. However, resistance proteins in the plant cells are still able to detect secreted T3E, thus activating the second layer of immune response, i.e., ETI. The identification of T3E by the plant R proteins results in HR [153]. Different types of defense responses can be elicited in the plant depending on the level and method of elicitor interaction. For example, hairpin elicitor proteins from *Erwinia amylovora* activate HR when introduced to the plant intercellular space; however, when sprayed, they cause the initiation of SAR [154,155].

Effectors released by the pathogen via T3SS can inhibit plant immunity and facilitate the colonization of the bacteria [156]. Plants can differentiate between the presence of microbes and pathogenesis patterns via the amplification of response to PAMPs by T3SS in bacteria [157,158]. LPS produced by Gram-positive and Gram-negative bacteria are capable of triggering plant immunity [28,159] (Table 1). Almost all bacterial cell walls have peptidoglycan, which provides the bacteria with its shape and gives the bacteria its structure [160]. For this reason, both plants and animals have developed peptidoglycan perception systems in order for them to be able to detect the presence of bacteria [161,162]. In fact, peptidoglycan acts as bacterial elicitor molecule in *Arabidopsis thaliana*, rice, and tobacco, where it elicits defense [28,104,163,164,165,166,167,168]. Another study also stated that Arabidopsis cells are not as sensitive as rice cells in the induction of PCD when stimulated by the LPS. This shows that different plants respond differently to LPS [169]. Through these studies, it has been established that when plants are induced with peptidoglycan, the immune response is activated, resulting in responses such as the increase of cytoplasmic calcium concentration and the accumulation of ROS [163,164].

Studies have reported that plants treated with LPS generate ROS [170,171] and express PR proteins as a consequence of the treatment [172,173]. Further, nitric oxide (NO) synthase was also induced when *A. thaliana* was treated with LPS, where NO plays a part in activating the defense genes and resistance against the pathogen [174]. In addition, LPS treatment has triggered SAR in many plant species [175,176,177]. The defense response is induced through the recognition of epitopes by different perception systems affected by LPS from different bacterial species [169,171,173,174,178,179,180,181,182,183].

Several studies have been conducted on the interaction between plants and flagellin. A study reported that flagellin-insensitive mutants were not responsive to bacteria as the *FLS2* receptor (contains transmembrane, cytoplasmic ser/thr kinase, and extracellular leucine-rich repeats (LRR) domains) involved in the recognition of flagellin was defective [150]. Following the binding of FLS2 to flagellin, the FLS2 protein complex undergo dynamic changes induced through the perception of the 22-amino acid epitope of flagellin (*flg22*), which is known as detergent-resistant membranes (DRMs) [20,184]. This results in the heterodimerization between FLS2 and its co-receptor, BRI1-associated kinase (BAK1) [153]. Somatic embryogenesis receptor-like kinase (SERK) is involved in FLS2 signaling, and BAK1 is a SERK member [185]. BAK1 is a better co-receptor for FLS2 compared to EFR [185] and is involved in the late endosome, where it is needed for FLS2 accumulation [44]. In fact, there is a sequence change in the mutant alleles of *FLS2,* which reinforces the fact that this gene plays a role in the flagellin-insensitive phenotype. From previous studies, it was reported that two hydrophobic domains at the N terminus between amino acids 1-23 and 815-831 are the signal peptides of transmembrane domains [186]. The process starts when the PRRs in plants recognize the flagellin produced by virulent and avirulent bacteria and initiate the immune response in plants through the production of ROS and ethylene and the activation of defense-related genes [151]. So, here, flagellin acts as the general conserved elicitor that initially activates the immune response of the host [187] (Table 1 and Figure 1).

### 5.3. Insect Elicitor Molecules: HAMPs and DAMPs vs. MAMPs

In addition, plants can also recognize specific components of insect saliva during herbivory. A plant’s response to damage by insect herbivores involves wound response and the recognition of certain insect-derived molecules [188,189,190,191]. Such insect-derived elicitor molecules can trigger signaling pathways, thereby initiating defense mechanisms in distant regions of the plants in anticipation of further damage. The herbivore-specific molecules that are secreted during insect feeding can be designated as herbivore-associated molecular patterns (HAMPs). These molecules participate in signaling and can induce defense reactions in plants. Other than components of oral secretion, these molecules include components of saliva and oviposition fluid [192]. Insect oral secretions have also developed into effectors that consist of specific proteins and chemicals that can inhibit the plant defense mechanisms. However, as time passes, some of the plants are able to overcome this inhibition when they have adapted themselves to recognize the molecules from the insect [193]. In other words, a lot of molecules in oral secretions can cause the plant to activate its defense response, involving enzymes such as glucose oxidase and β-glucosidase, peptides such as inceptin, and fatty acid conjugates such as volicitin [194].

One of the first types of elicitor identified in insect saliva was fatty acid–amino acid conjugates (FACs) or fatty acid amides [195] (Table 1). The well-studied FACs of *Manduca sexta* have a two-pronged approach where the volatiles produced can activate the defense response in a host whilst simultaneously attracting the predators of this caterpillar. Since this initial discovery, other types of elicitors have been identified where their specific elicitor molecule activity varies greatly among plant species [196]. Similarly, volicitin (N-(17-hydroxylinolenoyl)-L-glutamine) produced by *Spodoptera exigua* affected maize, which resulted in volatiles that functioned as attractants [195]. This chemical when applied to tobacco was shown to induce mitogen-activated protein kinase (MAPK), wound-induced protein kinase (WIPK), SA-induced protein kinase (SIPK), JA, SA, ethylene (ET), and JA–isoleucine conjugate (JA-Ile) [194]. The FACs produced induced the production of 7-epi-JA, which activates defense genes against insects [197]. In addition, inceptins and caeliferins in the oral secretions also activate defensive pathways to insects [43,198]. Furthermore, Tian et al. (2012) and Louis et al. (2013) reported on the induction of defense signaling in response to glucose oxidase (GOX) in the saliva of insects. The salivary component of *O. nubilalis* induces proteinase inhibitor 2 (PIN2) in maize and tomato [199,200]. However, there are some oral secretions that inhibit the defense pathway in plants. This has been reported in *Spodoptera littoralis* and *Pieris brassicae* where salivary secretions inhibited defense in order to enable larvae growth [201]. Therefore, these molecules can either activate or repress the plant defense responses [201,202], depending on which is leading the evolutionary process: the plant or the insect.

Some herbivorous insects may indirectly cause damage to plants, as these insects can generate damage-associated molecular patterns (DAMPs) that elicit a plant’s immune response [193]. For aphids to be able to use their effector proteins to manipulate or interrupt any process in plants, the proteins need to be secreted into the plant during plant–aphid interaction [43,203]. Elicitor molecules derived from aphids or any organisms that are associated with aphids might be responsible for controlling the PTI-induced responses in plants. These organisms inhabit the aphid and are capable of producing proteins that activate plant defense [204]. To date, only a handful of effector proteins associated with aphids have been identified and proven to be involved in inducing or repressing defense responses [204]. For example, Mp10 is essential for aphid fertility. This effector protein can also alter SA and JA-signaling molecules in plants and activate defense [205,206]. 

In addition to saliva, the honey dew produced by insects can also interact with the plants’ defense receptors [207]. These proteins sometimes carry bacterial proteins that include chaperones and flagellins that are produced by microbiota within the insect’s gut [208]. In insects such as aphids, pathogenic organisms such as *Pseudomonas syringae, Serratia marcescens*, and *Staphyloccocus* sp are part of their gut microbiota [208,209]. These bacterial proteins trigger plant immunity and can lead to the activation or suppression of defense responses, depending on the insect species [204]. 

As seen in Figure 1, the elicitor molecules produced by each group of organisms are distinct to that specific group. The mechanism of interaction and the scope of effect elicited by these proteins are largely dependent on the type of elicitor molecule. 

### 5.4. Non-Pathogenic Elicitor Molecules: Inducers of Systemic Resistance

Flagellin from the beneficial *Pseudomonas putida* WCS358 has been reported to induce systemic resistance against *P. syringae* in Arabidopsis [210]. Similarly, the LPS of certain Gram-negative bacteria have also been reported to induce systemic resistance. These tripartite amphipathic molecules with an immunologically active O-antigen side chain have induced ISR in *Pseudomonas fluorescens* pathosystems [210] and *P. putida* strains [210,211]. They have also been reported as effective ISR inducers in *Burkholderia cepacia–Phytophthora nicotianeae* interactions and *Rhizobium elti* G12-nematode infections of potato [177]. Further, siderophores produced by fluorescent Pseudomonas enable growth in iron-limiting environments [212]. Pyoverdines, a type of siderophores, behaves as a ISR elicitor [213,214]. WCS358 elicits ISR in Arabidopsis and tomato through siderophore [210,215,216]. SA is produced by some of these rhizobacteria that induce systemic resistance under iron-limiting conditions. Its role in the ISR elicitation process was demonstrated in the case of *Pseudomonas aeruginosa* KMPCH [217,218]. Nevertheless, several reports showed that SA production by other strains was not associated with ISR [211,219]. SA is also an intermediate in the biosynthesis of other siderophores such as pyochelin in *P. aeruginosa* [220]. *P. aeruginosa* 7NSK2-produced pyochelin is proposed to induce ISR in tomato [221,222].

Tri-N-alkylated benzylamine derivative (NABD) produced by *P. putida* BTP1 has ISR-inducing abilities [219]. Other researchers have reported the antibiotic properties of some Pseudomonas secretions such as pyocyanine and 2,4-diacetylphloroglucinol (DAPG), which induces systemic resistance [215,223,224]. Pyocyanine and pyochelin trigger ISR in *P. aeruginosa* 7NSK2-treated tomato [221]. *P. fluorescens* CHA0-produced DAPG stimulates ISR-mediated responses in *Peronospora parasitica* and *Meloidogyne javanica*-infected Arabidopsis and tomato [215,225]. In addition, biosurfactants such as rhamnolipids and lipopeptides produced by Pseudomonas and Bacillus were able to induce systemic resistance. This includes massetolide A, which protects tomato plants against *Phytophthora infestans* [226]. In another study, tomatoes overexpressing fengycins and surfactins from *Bacillus subtilis* 168 significantly induced resistance [227].

Other than the above-mentioned molecules, beneficial rhizobacteria produce exopolysaccharides [228] or *N*-acyl-L-homoserine lactone [229] as MAMPs. *Trichoderma virens*-produced hydrophobin-like elicitor Sm1 elicited ISR in maize. When cultivated with *Sm1* mutant strains, maize showed either increased or reduced protection, indicating a role for this elicitor in systemic resistance [230]. A similar response was produced by Trichoderma. The disruption of the *tex1* gene responsible for peptaibol production results in significantly reduced ISR in cucumber against *P. syringae* [231]. Contrary to the above, alamethicin, a peptaibol, resulted in the cell death of *A. thaliana,* indicating that these molecules may retain some phytotoxic element on certain species [232]. Recently, harzianolide and pentyl-pyranone have also been implicated as secondary metabolites that have a role in induced systemic resistance [233].

### 5.5. Non-Microbial Elicitors Molecules: Efficient Inducers of Defense

Biotic elicitors have also been isolated from algae, shrimp, and other crustaceous materials. Chitin and chitosan derived from algae or crustaceous material have been shown to induce defense mechanisms in plants through the binding of receptor molecules found in the plants’ plasma membrane [234,235]. Linear hepta-β-glucoside laminarin produced by brown algae elicits a defense in various plants species. A binding site has been identified for this elicitor molecule in rice and soybean [236,237,238]. These molecules, similar to pathogenic and non-pathogenic organisms, are able to mimic plant–pathogen response and activate SAR [239,240]. These non-microbial molecules successfully activate SA, JA, and systemin, leading to the activation and induction of systemic protection [241]. For example, in tomato, immediately after wounding by insects, prosystemin is synthesized, which then is proteolytically processed to systemin. Systemin binds to a cell surface receptor at the plasma membrane and activates defense. Chemical elicitors such as DL-β-aminobutyric acid (BABA) [242], 2,6-dichloro isonicotinic acid (INA) [243], benzo [1,2,3] thiadiazole (BTH) [240], and their derivatives are effective elicitors to induce the biosynthesis of plants.

It has been substantiated that BTH succeeded in inducing resistance to a lot of diseases, as it can be considered as a chemical that resembles SA [105]. For example, BTH and SA have been used as inducers in *Brassice juncea* (var. Rlm619), where it induces the production of enzymes such as peroxidase. It also prevents *Alternaria brassicae* from invading plants [244]. Moreover, BTH and humic acid (HA) in *G. max* minimized the risk of wilt disease and increased the growth rate of plants. The activity of oxidative enzymes is the highest when both elicitors, BTH and HA, are combined together [245]. These molecules are a good source for the exogenous induction of resistance in plant systems.

From the above elicitor systems, we can conclude that while there are a great variety of elicitors in the chemical and biological form, the downstream processes that lead to defense activation are similar. Most of these elicitors bind receptor molecules on the plant cell or interact within to elicit a defense response that results in ROS, HR, PCD, and the activation of resistance and defense-related genes *in planta* (Table 1; Figure 1).

**Table 1:** List of identified elicitor molecules of biotic stress agents in bacteria, fungi, insect, and synthetic molecules.

Origin	Elicitor Molecule	Effects Shown in	References
Bacteria	Harpin (HrpZ)	Various plants	[28,246,247]
	Flagellin	Most plants except rice (e.g., Arabidopsis)	[28,183]
	Cold shock proteins	Solanaceae	[28]
	Elongation factor (EF-Tu)	Brassicaceae	[28]
	Lipopolysaccharides (LPS)	Arabidopsis, pepper, and tomato	[28]
	Peptidoglycan	Arabidopsis and tobacco	[28]
	Oligogalacturonides	Arabidopsis and tomato	[108]
	Lipopeptides	Tomato	[248]
	Dimethylsulfide	Maize and tobacco	[249]
	Pseudobactin	Several plants	[214]
	Type-III secreted effector (T3E)	Several plants	[153]
	Tri-N-alkylated benzylamine derivative (NABD)	Bean	[219]
	2,4-diacetylphloroglucinol (DAPG)	Tomato, Arabidopsis	[211,219,220]
	Pyocyanine and pyochelin	Tomato	[221]
	Exopolysaccharides	Tobacco	[228]
	*N*-acyl-L-homoserine lactone	Tomato	[229]
Fungi	Β-glucans	Several plants (e.g., rice)	[28,250,251,252,253]
	Chitin/chitosan	Arabidopsis, rice, tomato, and wheat	[28,120,121,122,123,124]
	Cerebrosides A, C	Rice	[28,147]
	Ergosterol	Grapevine, tomato, and tobacco	[28,254,255]
	Xylanase	Tobacco and tomato	[28]
	HR-inducing protein	Rice	[256,257,258]
	PemG1	Arabidopsis and rice	[259,260]
	PebC1	Tomato	[55]
	Oligosaccharides	Rice	[107]
	Ethylene-inducing xylanase (EIX)	Several plants	[261,262,263,264]
Insects	Fatty acid amides in saliva	Several plants	[195]
	Glucose oxidase in oral secretion	Several plants	[194]
	β-glucosidase in oral secretion	Several plants	[194]
	Inceptin in oral secretion	Several plants	[194]
	Volicitin in oral secretion	Several plants	[194]
	Mp10 from aphids	Several plants	[194]
	Systemin	*S. peruvianum*	[265]
	PIP1	Arabidopsis	[266]
	Pep1–Pep6	Arabidopsis	[267,268]
	Rapid Alkalinization Factor (RALF) peptides	Arabidopsis	[269,270]
	Oligogalacturonides	Arabidopsis	[271]
	Extracellular ATP	Arabidopsis	[272]
Chemical	DL-β-aminobutyric acid (BABA)	Several plants	[242]
	2,6-dichloro isonicotinic acid (INA)	Bean	[243]
	Benzothiadiazole (BTH)	Several plants	[240]

## 6. Receptor Molecules: Perception and Activation

To date, only a few receptors that interact with elicitor molecules have been identified. The perception of elicitors involves surface-based receptors or intracellular receptors that will detect the pathogenic patterns. In most cases, the surface-based receptors are the primary detectors of pathogen-derived elicitors [28]. Receptor-like proteins (RLPs), receptor-like kinases (RLKs), and extracellular binding proteins constitute surface level multicomponent recognition complexes [273]. When specific receptor molecules in plants receive chemical signals released due to injury or pathogen invasion at any part of the plants, the receptor molecules are activated and they transduce the signaling cascade in plants, resulting in the activation of defense responses [192]. Table 2 provides a list of receptor molecules that have been reported thus far.

### 6.1. Fungal Receptor Molecules—Examples and Mechanism of Action

Chitin elicitor binding protein (CEBiP) is one of the receptor molecules involved in fungal chitin binding. This RLP is a LysM domain-containing protein and is essential in rice chitin signaling, which has been proven through studies that used rice cells with CEBiP–RNAi [34]. CEBiP is a chitin-specific receptor molecule, and its response and activity is not affected when other elicitor molecules such as LPS and peptidoglycan are present in the absence of chitin. CEBiP does not have an obvious intracellular domain and needs a partner such as an RLK to translate the perceived chitin signal into intracellular events [37,274]. A study proves that chitin elicitor receptor kinase (CERK, an RLK) and CEBiP form a receptor complex where CEBiP detects chitin elicitor molecules that are released in the plant, while OsCERK triggers the defense response by phosphorylating the proteins involved [275]. CEBiP does not have intracellular domains such as CERK and is present on rice cell membranes when the cell is infected by synthetic or pathogen-derived chitin and consequently causes the induction of defense response [190,276]. CERK is not only involved in rice immune systems but is also critical for chitin signaling in *A. thaliana* [36]. CERK1 along with AtLYK5 plays an important role in fungal chitin perception in *A. thaliana*, while OsCERK and OsCEBiP play a similar role in rice [127]. Insertional mutations in CERK block the induction of all chitin-responsive genes, resulting in greater susceptibility to the pathogens [37].

OsCERK and OsCEBiP have important roles in rice for membrane signaling and ligand binding. Both OsCERK and OsCEBiP are known as receptor complexes that form in plants. The formation of this complex is triggered by biologically active chitin oligosaccharides [275] with N-acetyl groups on both sides, which makes the LysM motifs bind to chitin fragments of OsCEBiP [277]. Usually for kinases, phosphorylation is required to trigger the activation of catalytic domains that enable the autophosphorylation of intracellular domains [278,279]. However, to date, OsCERK has not been reported to produce dimers [280], although the chitin binding activity of OsCEBiP results in dimerization. The ability of specific mutants to bind chitin fragments does not correlate with dimerization [127]. In the “sandwich-like” model reported by Squeglia et al. (2017), chitin induced the dimerization of OsCEBiP, resulting in OsCERK being closer for the autophosphorylation of receptor kinase to take place. Acetyl moieties of chitin mediate the cross-link operation that dimerizes OsCEBiP molecules [278].

LYP4 and LYP6 are CEBiP-like proteins that are equally reactive to peptidoglycan and chitin [166]. The reactivity of these two proteins toward chitin elicitor is low when the expression of the proteins are low, but there is no clear explanation on the relationship between LYP4/6 and CEBiP or OsCERK [274]. Researchers have reported that the level of H_2_O_2_ generated by CEBiP mutant, *cebip,* is lower than that observed in non-transformed rice cells. There is also no obvious change in the level of H_2_O_2_ when *cebip* is treated with peptidoglycan and LPS. A study showed that there is almost no change in the CEBiP expression, H_2_O_2_ generation, and reaction to the chitin elicitor detected in the *cebip*-cultured cells, which indicates that the chitin elicitor response relies on CEBiP [274,281]. Results from the affinity labeling bespeaks that CEBiP is important in binding to the chitin elicitor in cultured cells and leaves. Studies show that *cebip*-cultured cells react the same way as wild-type cells in response to peptidoglycan and LPS. As LYP4 and LYP6 engage in the identification of both peptidoglycan and the chitin elicitor, it is not as specific as CEBiP [166].

Although CEBiP is an important chitin elicitor binding protein involved in signaling and the perception of chitin elicitors, any changes to the gene itself do not result in significant change to immunity against rice blast fungus. This indicates that the recognition of chitin-derived oligosaccharides between rice and *M. oryzae* is limited. This might be due to the competition between LysM Protein 1 (Slp1) that is secreted by *M. oryzae* and CEBiP in binding to chitin oligosaccharides to repress the chitin elicitor that induces the defense response [282]. Chitin fragments have been identified to activate several defense responses.

In addition, another study found that ethylene-inducing xylanase (EIX), a fungal protein, initiates ethylene biosynthesis, HR, and PR proteins expression in a variety of plants [261,262,263,264]. EIX mutants without enzymatic activity have retained the ability to elicit HR, indicating that HR elicitation does not depend on xylanase activity [283,284,285]. Tomato and tobacco have been used as responding cultivars, where it has been reported that EIX binds specifically to the plasma membrane of both cultivars [286]. There are two members in LeEix locus in tomato that have been characterized: *LeEix1* and *LeEix2*. An experiment was conducted to show the potential of *LeEix1* and *LeEix2* to bind EIX where both can re-establish the binding to the EIX elicitor, even though only *LeEix2* shows positive results in transmitting the signals that are needed to activate HR [145]. There are direct interactions between *LeEix2* and EIX elicitor where other plant proteins are not needed in the binding of these two proteins. Transgenic plants treated with *LeEix2* bind to the EIX elicitor, and *LeEix2* expressed in the COS-7 cells indicates its function as a receptor to the EIX elicitor [145]. *LeEix1* is unable to transmit the signal needed to initiate HR, as there is a difference in its cytoplasmic domain compared to *LeEix2*. The interaction between the EIX elicitor and *LeEix2* initiates receptor-mediated endocytosis [145].

In a separate study, two LRR–RLPs that respond to fungal EIX, *S1Eix1* and *S1Eix2*, were found in tomato. EIX acts freely as an elicitor of defense response in tomato and tobacco without depending on endoxylanase activity [145,284]. *S1Eix1* and *S1Eix2* have a different role, even though both bind to EIX and have 81.4% similarity. A study proposed that *S1Eix1* obstructs the signal for plant defense and plant cell death when it interacts with EIX, since *S1Eix1* receptors are deprived of signaling from EIX. *S1Eix2* attributes toward conveying signals to actuate the plant immunity [145,287].

### 6.2. Bacterial Receptor Molecules—Examples and Mechanism of Action

Several studies have identified bacterial receptor molecules that recognize bacterial conserved PAMPs. To begin with, LysM was first recognized in bacteriophages and bacterial proteins. Now, this motif is found to mediate the binding of GlcNAc-containing molecules in addition to being found in peptidoglycan binding proteins [168]. CERK1 is important for peptidoglycan recognition in *A. thaliana* and rice [288,289]. However, some bacterial effectors have been reported to restrict CERK1-mediated signaling [290,291].

In addition, there are other resistance genes that encode receptor molecules involved in plant defensive mechanisms that contribute toward plant resistant phenotypes toward bacteria. These genes include *RPS2*, [292,293], *Pto* [294], *Xa21* [295], and *Cf9* [296] (Table 2). *RPS2*, *Pto*, and *Cf9* provide narrow-spectrum resistance, while *Xa21* provides multi-spectrum resistance to *Xanthomonas oryzae* pv. *oryzae*. *Xa21* is a kinase that contains non-arginine-aspartate (non-RD) motif and LRR in the extracellular domain [297], which provides the ability to identify LPS [298,299].

Furthermore, the involvement of FLS2 in the identification of flagellin is proven by ectopic expression, where the relationship between flagellin and the expression level of FLS2 results in the activation of the defense system [183]. The C-terminal region of the FLS2 consists of putative protein kinase catalytic domains [300]. The binding of flagellin caused the activation of kinase, which led to the phosphorylation of the target. The *Xa21* in rice has a similar gene structure to FLS2 [295], while *Cf-2.1* and *Cf-2.2* (tomato resistance genes) [301] and TOLL like receptors (TIR) are similar to the extracellular domain of FLS2 [302]. Studies show that when plants are treated with flg22, MAMP-mediated immunity is triggered, and plants are protected against pathogens. As the stomata is the common entry for bacteria, stomatal closure occurs upon flagellin perception in plants [303]. The absence of FLS2 in plants makes it more vulnerable to infection, as FLS2 plays a role in early infection where it prevents bacterial entry before it gets into the apoplast [31]. In addition, flg22 also triggers delayed nodule organogenesis in the formation of early symbiosis between *Lotus japonicus* and *Sinorhizobium meliloti* [20]. The MAMP trigger immunity (MTI)-suppressing factors might have been secreted into the plants, as there is no consequence of flg22 detected once symbionts are formed [20,304]. However, the proteins that are involved in the identification of different pathogen signal molecules are highly conserved, which shows that similar mechanisms play a role in the interaction and the activation of the defense response against a diverse array of pathogens.

### 6.3. Insects Receptor Molecules—Examples and Mechanism of Action

Insect feeding leads to the rapid accumulation of protease inhibitors throughout the plant, even in undamaged areas far from the initial feeding site [92,305]. Plants recognize insect elicitors that initiate defense response through the activation of kinases and phytohormone networks [104,190,191,306,307,308]. JA, SA, and ET are the main players in chewing and phloem feeding insect herbivory [305,309]. There are several DAMPs and HAMPs peptides that are available post-processing during insect herbivory [310,311]. A study reported that insects produced prosystemin that lacked N-terminal leader sequence, indicating a passive systemin release [312]. The systemin cell surface receptor from tomato is an LRR receptor kinase, and the binding of systemin to its receptor initiates an intracellular signaling process that results in the activation of JA biosynthesis and accumulation [313]. In target tissues, JA activates the expression of genes that encode protease inhibitors. Since the initial discovery of systemin, many systemin-like signaling peptides have been identified in tomato that have a role to play in pests and pathogens defense [314]. AtPep1–AtPep8 and ZmPep1 are two well-characterized DAMPs in *A. thaliana* and maize, respectively [311]. Unlike prosystemin, Pep epitopes are in the C-terminus of PROPEP, and these epitopes recognize class XI LRR–RLKs PEPR1/PEPR2 in *A. thaliana*. The Pep–PERR complex activates broad-spectrum resistance against pathogens, nematodes, and insects [22,315,316,317,318]. In addition, the lack of an N-terminal on PROPEP implies that this protein is released through membrane damage [319]. Since PEPR is required for DAMPs’ response, the PROPEP most probably has to go through processing to generate active Pep and the activation of defense [316]. Interestingly, Peps have been reported to exhibit family-level diversification, as observed in angiosperms that result in recognition specificities [315,320].

**Table 2:** List of identified receptor molecules in plants against bacteria, fungi, and insects.

Gene	Plant	Pathogen	References
*FLAGELLIN SENSITIVE2 (FLS2)*	*Arabidopsis thaliana*	Bacteria	[183]
*Xanthomonas oryzae resistance 21 and 26 (Xa21 and Xa26)*	*Oryza sativa*	Bacteria	[295,321]
*Ceramide kinase (CERK)*	*Arabidopsis thaliana, Oryza sativa*	Bacteria and Fungi	[275]
*Chitin elicitor binding protein (CEBiP)*	*Arabidopsis thaliana*	Fungi	[34]
*EF-Tu Receptor (EFR)*	*Arabidopsis thaliana*	Bacteria	[30]
*Hypernodulation aberrant* *root formation (HAR1)*	*Lotus japonicas*	Bacteria	[322]
*Brassinosteroid LRR receptor kinase (CURL3)*	*Solanum esculentum*		[265]
*Ribosomal protein S2 (RPS2)*	*Arabidopsis thaliana*	Bacteria	[292,293]
*Resistance to P. syringae (Pto)*	*Solanum lycopersicum*	Bacteria	[294]
*Cladosporium fulvum resistance protein 9 (Cf9)*	*Solanum lycopersicum*	Fungi	[323]
*Systemin Receptor (SYR1)*	*Solanum lycopersicum*	Insect	[324]
*Cold Shock Protein Receptor (CORE)*	*Solanum lycopersicum*	Bacteria	[325]
*RECEPTOR-LIKE PROTEIN REQUIRED* *FOR CSP22 RESPONSIVENES (CSPR)*	*Nicotiana benthamiana*	Bacteria	[326]
*LIPOOLIGOSACCHARIDE-SPECIFIC REDUCED ELICITATION (LORE)*	*Arabidopsis thaliana*	Bacteria	[327]
*Receptor like protein 23 (RLP23)*	*Arabidopsis thaliana*	Fungi	[328]
*Receptor like protein 42 (RLP42)/ RESPONSIVENESS TO BOTRYTIS POLYGALACTURONASES1 (RBPG1)*	*Arabidopsis thaliana*	Fungi	[329]
*Lysin motif protein 1,3,2 (LYM1, LYM3, LYM2)*	*Arabidopsis thaliana*	Bacteria and fungi	[288,330]
*PEP receptor 1 and 2 (PEPR1/PEPR2)*	*Arabidopsis thaliana*	Insect	[22,267,268,315,316,317,318]
*Systemin receptor 160 (SR160)*	*Solanum peruvianum*	Insect	[265]
*Wall-associated kinase 1 (WAK1)*	*Arabidopsis thaliana*	Bacteria and fungi	[271]
*S-receptor-like kinase (SRLK) like gene (I-3/I)*	*S. pennelli, S. pimpinellifolium*		[331,332]
*Receptor like protein 30 (RLP30)*	*Arabidopsis thaliana*	Bacteria and fungi	[333]
*Receptor like protein 1 (RLP1)*	*Arabidopsis thaliana*	Bacteria	[334]
*Lysin motif receptor kinase 5 (LYK5)*	*Arabidopsis thaliana*	Fungi	[335]
*Resistance to Leptosphaeria maculans 23 (RLM/LepR3)*	*Brassica napus*	Fungi	[336,337]
*Cladosporium fulvum resistance protein 2/4/5/9 (Cf-2/4/5/9)*	*Solanaceae*	Fungi	[114,301,323,338,339]
*Resistance gene homologue Cf-4 (Hcr9-4E)*	*Solanum hirsutum*	Fungi	[340,341]
*Does not Respond to Nucleotides1 (DORN1)*	*Arabidopsis thaliana*	Bacteria	[272]
*Lysin Motif-containing Proteins 4 and 6 (LYP4 and LYP6)*	*Oryza sativa*	Bacteria and Fungi	[166]
*Secreted LysM Protein1 (Slp1)*	*Oryza sativa*		[282]
*Ethylene-inducing xylanase receptor 1 and 2 (LeEix1 and LeEix2)*	*Solanum lycopersicum*	Fungi	[262]
*Tomato Ethylene-inducing xylanase receptor 1 and 2 (S1Eix1 and S1Eix2)*	*Solanum lycopersicum*	Fungi	[262,284,287]

### 6.4. Variations in Elicitors Affect Receptor-Binding Affinity

Elicitor molecules produced by symbiotic and infectious organisms vary structurally, which enables the host to discriminate between them and provide specialized perception at the plant’s cell wall. For instance, the natural occurring differences in pyoverdins produced by WCS358, WCS374, and CHA0 results in the variation of peptide chains produced, which affects the perception of the molecule at the host cell level [214]. NABD, SA, DAPG, pyocyanin, or volatile 2,3-butanediol as determinants of ISR do not show any signs of obvious structural similarities. However, comparing NABD and pure benzylamine indicates that the aromatic amino part of the molecule affects the biological function of these determinants [227]. Similarly, SA and DAPG have an aromatic phenolic group that is recognized by specific plant cell receptors. However, lipopeptides contain a less specific mechanism in binding to receptors, as their amphiphilic nature enables LP to insert itself into the lipid bilayer and create channel imbalances in the membrane [342].

In fungi and bacteria, there are two different types of receptor molecules: one that is specific to the taxa of organism and one that is open to both fungi and bacteria. Receptor interaction is largely controlled by the type of elicitor and the ability of the receptor molecule to bind these elicitors. However, there is a difference between microorganisms and insect interaction with regard to receptor molecules, as the protein inhibitors produced by the insects are transmitted directly into the cell through feeding without the need for plant cell surface receptors. Nonetheless, the cascades triggered thereafter are similar with a slight variation in the genes activated between insects and pathogens (Figure 1; Table 2).

## 7. Future Prospects and Conclusions

The interaction between receptor and elicitor molecules provides understanding of the molecular identification, cell biology, and the evolution of the process of defense activation between kingdoms. A thorough study on the function of the plant immune system will support crop development for food safety and security. Given a better understanding of the immune system, researchers will be able to engineer plants with enhanced resistance to disease and pest. Focus is especially on identifying the pathways that activate plant defense in order to improve disease and pest resistance. By understanding the interaction between the elicitor and receptor, researchers can enhance the existing defense system in plants to ‘fight’ against the invading organism. Here, we list some of the possible applications for the information obtained.

(1) **Engineering of crops with disease and pest resistance:** Genetic engineering and plant breeding may utilize specific quantitative trait loci (QTLs) or loci with key resistance genes to generate or select for new varieties. These lines may also act as donors in diseases and pest resistance breeding.
(2) **Elicitor molecules as triggers of defense:** Elicitors from non-virulent strains or attenuated virulent strains may be utilized to trigger the immune response of plants and activate defense signaling that involves SA, ethylene, and JA-dependent/ independent pathways.
(3) **Identifying simulating signaling molecules:** Inducers such as acibenzolar-S-methyl (ASM) or benzothiadiazole (BTH) are able to activate SAR [105]. Therefore, the identification of various other molecules with similar tendency is encouraged.
(4) **Elicitors from beneficial rhizobacteria:** The identification of rhizobacterial species with the tendency to secrete chemical components that activate defenses such as LPS, flagellins, siderophores, and SA will be beneficial as biocontrols and plant growth regulators.
(5) **Elicitors from beneficial fungi and yeast:** Requires the identification of more fungal and yeast strains that are able to generate antimicrobial properties as well as function to induce ISR.
(6) **Still searching for receptors:** To date, the molecular level activation of defense in response to PAMPs, DAMPs, HAMPs, and MAMPs has not been extensively studied and reported [343]. While the interaction with PAMPs and MAMPs results in activated defenses, there has to be some distinction at the molecular level, as the plant shelters non-pathogenic/beneficial microbes (MAMPs) in the interaction versus antagonistic reaction against pathogens (PAMPs). In addition, plant immunity triggered by MAMPs is based on priming with no reprogramming and fitness cost, while PAMPs results in the direct activation of the arsenal. To date, more plasma membrane receptor molecules have been identified for PAMPs, while MAMP-related receptors have not been reported. In theory, MAMPs would also require some form of binding site for the activation of the defense response [34,145,318,344,345,346]. The same is true of DAMPs and HAMPs, where little is known on receptors and the receptor–elicitor interactions.

Furthermore, a more extensive dissection of the interaction between elicitor receptor molecules is required to (1) differentiate the responses in a cell surface level versus cytoplasmic receptor system, (2) identify the differences in response to fungi, bacteria, nematode, insects, and other biotic components in a elicitor–receptor complex, (3) discriminate between the specific versus nonspecific response of receptors to elicitor or elicitor-like molecules, (4) elucidate if there is a difference between the defense response mounted and the key players in systems with specific versus nonspecific receptors, (5) identify and differentiate at the molecular level the function of PAMP, MAMP, DAMP, and HAMP receptors, and finally (6) critically evaluate the difference between SAR and ISR at the molecular level with specific regard to elicitor–receptor pairings. Through a systematic dissection of the above, we hope that we are able to further understand the complexities involved in the role played by the receptor–elicitor complex in mounting the defense response in plants.
